# Miscellany

**Published:** 1912-03

**Authors:** 


### MISCELLANY

---

China sends to the United States annually, about thirty million pounds of tea and 15 or 25 million pounds of rice.

At a time when western New York is piled deep with snow, Atlantic City is available for open-air life. See the advertisement of the Chalfonte.

